# When do confounding by indication and inadequate risk adjustment bias critical care studies? A simulation study

**DOI:** 10.1186/s13054-015-0923-8

**Published:** 2015-04-30

**Authors:** Michael W Sjoding, Kaiyi Luo, Melissa A Miller, Theodore J Iwashyna

**Affiliations:** Department of Internal Medicine, The Division of Pulmonary & Critical Care Medicine, University of Michigan, 3916 Taubman Center, 1500 E. Medical Center Dr., SPC 5360, Ann Arbor, MI 48109-5360 USA; College of Literature, Science and the Arts, University of Michigan, Ann Arbor, MI USA; VA Center for Clinical Management Research, Ann Arbor, MI USA; Institute for Social Research, Ann Arbor, MI USA; Department of Epidemiology and Preventive Medicine, Australian and New Zealand Intensive Care Research Centre, Monash University, Melbourne, VIC Australia

## Abstract

**Introduction:**

In critical care observational studies, when clinicians administer different treatments to sicker patients, any treatment comparisons will be confounded by differences in severity of illness between patients. We sought to investigate the extent that observational studies assessing treatments are at risk of incorrectly concluding such treatments are ineffective or even harmful due to inadequate risk adjustment.

**Methods:**

We performed Monte Carlo simulations of observational studies evaluating the effect of a hypothetical treatment on mortality in critically ill patients. We set the treatment to have either no association with mortality or to have a truly beneficial effect, but more often administered to sicker patients. We varied the strength of the treatment’s true effect, strength of confounding, study size, patient population, and accuracy of the severity of illness risk-adjustment (area under the receiver operator characteristics curve, AUROC). We measured rates in which studies made inaccurate conclusions about the treatment’s true effect due to confounding, and the measured odds ratios for mortality for such false associations.

**Results:**

Simulated observational studies employing adequate risk-adjustment were generally able to measure a treatment’s true effect. As risk-adjustment worsened, rates of studies incorrectly concluding the treatment provided no benefit or harm increased, especially when sample size was large (n = 10,000). Even in scenarios of only low confounding, studies using the lower accuracy risk-adjustors (AUROC < 0.66) falsely concluded that a beneficial treatment was harmful. Measured odds ratios for mortality of 1.4 or higher were possible when the treatment’s true beneficial effect was an odds ratio for mortality of 0.6 or 0.8.

**Conclusions:**

Large observational studies confounded by severity of illness have a high likelihood of obtaining incorrect results even after employing conventionally “acceptable” levels of risk-adjustment, with large effect sizes that may be construed as true associations. Reporting the AUROC of the risk-adjustment used in the analysis may facilitate an evaluation of a study’s risk for confounding.

**Electronic supplementary material:**

The online version of this article (doi:10.1186/s13054-015-0923-8) contains supplementary material, which is available to authorized users.

## Introduction

Financial, ethical, and practical constraints prevent randomized clinical trials (RCTs) from being conducted in many cases to guide clinical decision-making. The opportunity for observational studies to fill in these evidence gaps may be increasing, as routinely collected patient data become more detailed [1] and National Institutes of Health-sponsored clinical trial data are now publicly available for secondary use [2,3]. In the ICU in particular, the volume of routinely collected patient data available for analysis is staggering in size and scope [4,5]. As data collection and computation becomes cheaper, the role of observational studies in clinical medicine is unlikely to diminish [6].

Confounding is a particular threat in observational studies when comparison groups are different because of so-called non-random allocation, because patients are given therapies doctors think are best for them, rather than because of a coin flip [7,8]. For critically ill patients, these treatment choices are frequently informed by a patient’s severity of illness, and observational studies assessing the effect such treatments are at risk of obtaining incorrect results due to confounding by indication. If a patient’s indication to receive treatment is their higher severity of illness compared to those who do not receive treatment, a spurious treatment-outcome association may be measured solely due to confounding by severity of illness. Adjusting for severity of illness within statistical regression is possible [9], but whether such adjustment succeeds at removing these baseline differences between patient groups is often not clear. To overcome confounding, sophisticated severity of illness risk-adjustors with area under the receiver operator characteristic curve (AUROC, a common measure of accuracy) as high as 0.8 to 0.9 have been developed for ICU patients [10-13]. Unfortunately, these same scores often display AUROCs of 0.7 to 0.8 in external validation, may be even lower in situations of particular clinical interest [14,15], and are sometimes replaced by even less accurate comorbidity adjustment scores such as the Charlson and Elixhauser. Although imperfect risk adjustment and residual confounding are universally acknowledged in the limitations sections of observational studies, there is often little effort to assess their likelihood or the magnitude of such effects.

Because there are not widely implemented techniques to assess whether observational studies are valid when there is risk of confounding, the current study seeks to clarify and provide guidance to address this problem. We simulated a series of observational studies that replicate the common scenario in the ICU, where one is interested in determining whether a treatment has an independent effect on mortality, when it is also true that more severely ill patients are more likely to receive the treatment. We simulated studies in which a treatment had no direct effect on mortality, and thus, was safe to administer to critically ill patients, as well as scenarios in which the treatment provided a truly beneficial effect on mortality. We tested the hypothesis that over a range of worsening risk adjusters, observational studies would be increasingly likely to make an incorrect conclusion about the treatment’s true effect, thus, making them unreliable as evidence to inform clinical practice. We sought to develop intuitions for assessing risk of obtaining such results under various scenarios in observational studies, and to quantify the magnitude of apparent associations that can be measured solely due to confounding.

## Methods

We performed Monte Carlo simulations of observational cohort studies where an investigator evaluates the independent effect of a hypothetical treatment (for example, drug, procedure, or sepsis bundle), when the treatment was more often given to patients at higher risk of death compared to patients not receiving the treatment. During each simulated study, we drew a random sample of patients from a hypothetical cohort of patients receiving non-surgical mechanical ventilation. This hypothetical population’s risk distribution was modeled after non-surgical mechanical ventilation patients hospitalized at US Department of Veterans Affairs hospitals [13]. In a sensitivity analysis presented in the Appendix, we generated a hypothetical population designed to model situations where clinicians can estimate with good accuracy which patients would live or die. This population had a bimodal distribution of risk: most patients were assigned a relatively low probability of death (clinicians felt they would live) but a small proportion was assigned a high probability (clinicians felt they would likely die). Because literature suggests physicians can predict mortality with AUROCs of approximately 0.85, by construction, this risk distribution was modeled with a similar baseline predictive ability [16].

### Generation of the risk adjuster

We varied the accuracy of the risk adjuster available to the investigator to adjust for differences between patients. We set patients’ baseline risk of death as their true risk in each simulation, and created a risk adjuster that could predict their risk of death with varying accuracy. We varied the risk adjuster accuracy by adding random error to each patient’s baseline risk, and quantified its accuracy by calculating the risk adjuster AUROC for predicting death in the sample (these AUROCs represent in-sample accuracies, not external accuracies). The AUROC was calculated by post-estimation after fitting a logistic regression with the risk adjuster as the exposure and death as the outcome.

### Development of low and high confounding scenarios

We varied the degree of confounding across simulated studies. Although treatment receipt was randomly assigned, to introduce confounding we weighted the assignment based on the patient’s baseline risk of death. By more heavily weighting patients’ baseline risk, we could increase the level of confounding. We developed two hypothetical scenarios of interest to best approximate situations likely to occur in clinical practice, a scenario where the administration of the treatment was mildly confounded, and a scenario of high confounding.

In low confounding scenarios, patients at the 10^th^ percentile of risk of death (15% chance of death) received the treatment 30% of the time, while patients at the 90^th^ percentile of risk (75% chance of death) received the treatment 60% of the time. Thus, compared to low-risk patients, patients at the highest risk of death were approximately twice as likely to receive the treatment when confounding was low.

In the high confounding scenarios, a low-risk patient had a low chance of receiving the treatment, but the chance rose rapidly as risk increased. Patients at the 10^th^ percentile of risk received the treatment 15% of the time, while patients at the 90^th^ percentile of risk received the treatment 85% of the time. Thus, compared to low-risk patients, patients at the highest risk of death were approximately five times as likely to receive the treatment when confounding was high.

### True treatment effect

We set the treatment’s true effect on mortality during each simulation. Because observational studies often employ logistic regression to adjust for differences between groups, we expressed the treatment’s true effect as an odds ratio (OR). We created scenarios in which the treatment had no effect on mortality (OR = 1.0), as well as scenarios where the treatment was truly beneficial (OR = 0.6 or 0.8). When the treatment was beneficial, patients receiving the treatment had 0.6 or 0.8 times the odds of death compared to prior to receiving the treatment. To more easily conceptualize ORs, we calculated the risk difference after treatment and graphically illustrated the shift in the distribution of risk before and after treatment.

### Simulation analysis

We varied four parameters during simulations: the number of patients in the study, the risk adjuster accuracy, the degree of confounding, and the true treatment effect. We sampled with replacement either n = 1,000, or n = 10,000 cases. To determine whether a patient died during each simulation, we performed a Bernoulli trial for each patient, setting the patient’s risk of death as the probability of a positive trial result. For patients who did not receive the treatment, their risk of death was set to their baseline risk. For patients who were randomly assigned to receive the treatment, their risk was adjusted based on the treatment’s true effect.

To determine the treatment’s measured effect during each simulation, a logistic regression of the treatment on death was performed, using the risk adjuster to control for confounding. Guided by the measured treatment effect and statistical significance of the result, false negative rates and false harm rates were calculated for each set of simulation parameters. False negative studies incorrectly concluded that the treatment had no effect on mortality (*P*-value >0.05) when the treatment was truly beneficial (true OR <1.0). False harm studies incorrectly concluded that treatment’s odds of mortality were statistically significantly greater than 1.0 (*P*-value <0.05) when the treatment was either safe (true OR = 1.0) or provided true benefit (true OR <1.0).

We calculated the average measured OR for each set of simulations to present the relationship between risk adjuster accuracy and measured treatment effect. We also described the range of ORs possible for each set of parameters, presenting the median, intra-quartile, and 95^th^ percentiles in box and whisker plots [17]. All data management and simulations were conducted in Stata 13 (College Station, Texas). The sample code for this analysis is available in Additional file 1 in the online supplementary material. This simulation work did not use individual patient data, nor involve any interaction with patients. As such, it did not require ethical approval or require any patient consent.

## Results

**Figure 1:**
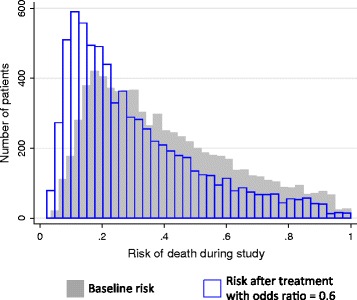
Distributions of risk of death among the patients used in the Monte Carlo simulations. The gray distribution represents the baseline risk of death while the blue distribution represents the risk after administration of a treatment with an odds ratio of 0.6 for mortality.

The distribution of baseline risk for patients receiving non-postoperative mechanical ventilation, as well as the distribution of risk after treatment with a treatment with an OR of 0.6 is shown in Figure 1. For a patient with the median baseline risk, receiving a treatment with an OR of 0.6 would decrease their risk of death from 35% to 24%, while receiving a treatment with an OR of 0.8 would decrease their risk from 35% to 30%. In an unadjusted analysis, if the true treatment effect (OR) was 0.6, studies measured the treatment’s effect accurately when no confounding was present, but measured the effect as 1.0 with low confounding and 1.6 with high confounding. Similarly, when the treatment effect (OR) was 0.8, unadjusted analysis of studies with no confounding measured the effect accurately, but measured the effect as 1.3 with low confounding and 2.0 with high confounding.

**Figure 2:**
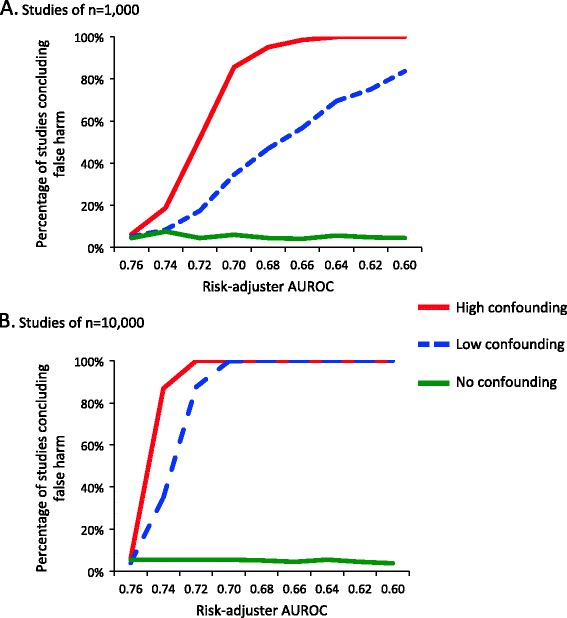
Rates of falsely concluding a safe treatment (odds ratio = 1.0) caused statistically significant harm among simulated cohort studies. **(A)** Rates in studies of n = 1,000 and **(B)** rates in studies of n = 10,000. AUROC, area under the receiver operator characteristic curve.

In simulated studies of safe treatments (true OR = 1.0), studies could make one of two conclusions: correctly conclude the treatment had no association with mortality or incorrectly conclude the treatment was harmful. When confounding was absent, studies concluded the treatment was harmful 5% of the time, which was expected because the threshold for statistical significance (*P*-value) was set at 0.05 (Figure 2A and B). The rates of studies detecting a false harm increased as the risk adjuster accuracy decreased, especially in high confounding scenarios. Using a risk adjuster with an AUROC of 0.70, approximately half of studies of n = 1,000 would report a statistically significant harm for a truly safe treatment due to residual confounding by indication in the low confounding scenarios, rising to nearly 90% if there was high confounding. When study size was 10,000, however, studies detecting false harm rapidly increased to 100% in all scenarios (Figure 2B). In these data, risk adjusters with an AUROC of 0.76 were generally protected from obtaining incorrect results.

**Figure 3:**
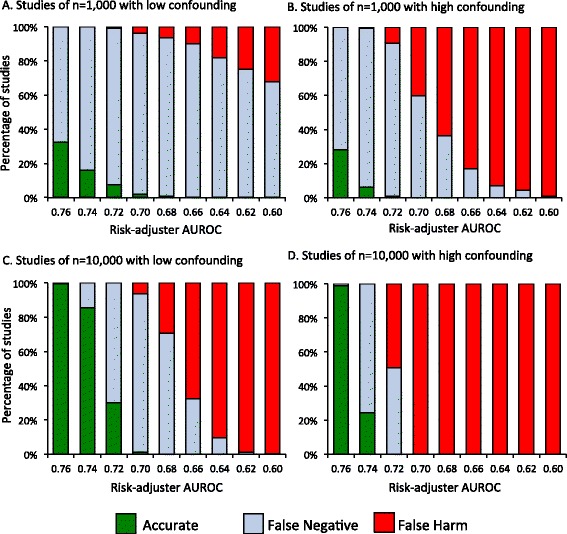
Rates of falsely concluding a beneficial treatment (odds ratio = 0.8) caused no benefit (false negative) or statistically significant harm (false harm) among simulated cohort studies. **(A)** Rates in low confounding scenarios of n = 1,000. **(B)** Rates in high confounding scenarios of n = 1,000. **(C)** Rates in low confounding scenarios of n = 10,000. **(D)** Rates in high confounding scenarios of n = 10,000. AUROC, area under the receiver operator characteristic curve.

In simulated studies of truly beneficial treatments (true OR of 0.6 or 0.8), studies could make one of three conclusions: the treatment had a statistically significant benefit, the treatment had no effect (false negative), or the treatment had a statistically significant harm (false harm). If the true treatment effect was an OR of 0.8, studies with a sample size of 1,000 had high false negative rates in low confounding scenarios, even with good risk-adjustment (Figure 3A). In high confounding scenarios, studies had high false negative rates when risk-adjustment was good but high false harm rates when risk adjustment was poor (Figure 3B). Studies of 10,000 patients with good risk adjustment were generally able to detect the treatment’s beneficial effect (Figure 3C). However, as risk adjustment worsened, false negative rates rose quickly in studies of 10,000 patients in both low and high confounding scenarios, followed by increasing rates of detecting false harm (Figure 3C and D).

**Figure 4:**
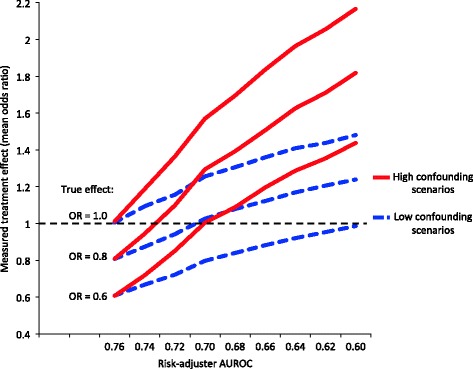
Relationship between the true treatment effect, risk-adjuster accuracy, and measured treatment effect in low and high confounding scenarios. AUROC, area under the receiver operator characteristic curve.

Because readers not only consider the statistical significance but also the magnitude of the OR when interpreting a result, the association between the risk adjuster accuracy and the measured ORs are shown in Figure 4. In all confounded studies, the average measured OR increased as risk-adjuster accuracy decreased, although this rise was faster when confounding was high. When the treatment’s true effect was an OR = 0.6, the mean measured OR crossed 1.0 when the AUROC was 0.60 in low confounding scenarios, but crossed 1.0 when the AUROC was 0.70 in high confounding scenarios. When the treatment’s true effect was an OR = 0.8, the mean measured OR crossed 1.0 when the AUROC was 0.70 in low confounding scenarios, but crossed 1.0 when the AUROC was 0.72 in high confounding scenarios.

**Figure 5:**
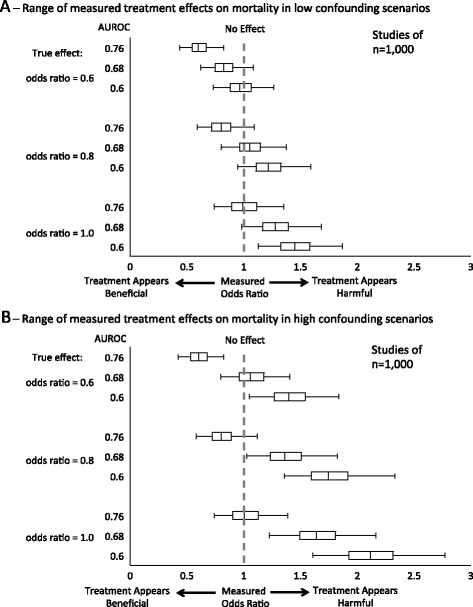
Distribution of measured effect sizes in simulated studies when sample size is 1,000. The center line of the box represents the 50^th^ percentile odds ratio, the box extends from the 25^th^ percentile to the 75^th^ percentile, and whiskers extend from the 2.5^th^ and 97.5^th^ percentile (representing 95% confidence intervals). **(A)** Low confounding scenarios. **(B)** High confounding scenarios. AUROC, area under the receiver operator characteristic curve.

Figure 5 depicts the range of measured treatment effects among simulated studies of 1,000 patients, including the interquartile range and 95% interval estimates. When confounding was low and risk adjustment was poor (AUROC = 0.6), the 95% interval range of measured ORs was 0.73 to 1.26 when the treatment’s true OR = 0.6, 0.95 to 1.59 when the treatment’s true OR = 0.8, and 1.12 to 1.87 when the treatment’s true OR = 1.0 (Figure 5A). In high confounding scenarios, the 95% interval range of the estimate was both wider and higher. When risk adjustment was poor (AUROC = 0.6), the 95% interval range was 1.05 to 1.83 when the true OR = 0.6, 1.34 to 2.33 when the true OR = 0.8, and 1.61 to 2.77 when the true OR = 1.0 (Figure 5B).

Finally, when we repeated the analysis in the cohort of patients with a bimodal distribution of risk, the key difference in the results was the level of risk adjustment required to protect against obtaining inaccurate results. While an AUROC of 0.76 protected studies using the mechanical ventilation cohort, the bimodal distribution required an AUROC of 0.85 in large studies with high confounding (results provided in Additional file 1 in the online supplement, Table S3, Table S4, Figure S1, Figure S2).

## Discussion

In the current study, we simulated observational studies that measured the association between a hypothetical treatment and mortality, when confounding by severity of illness was present. Although we identified AUROC values that protected against obtaining incorrect results, the accuracy of risk-adjustment necessary depended on the population studied, study size, and degree of confounding. In scenarios where the hypothetical treatment had no association with mortality, or scenarios when the treatment was truly effective, studies were frequently unable to measure its true effect. Studies often concluded the treatment caused statistically significant and clinically meaningful harm as a result of inadequate risk adjustment. Finally, our study illustrated how larger sample size increases the risk of confounding if larger sample sizes are not accompanied by better risk adjustment.

There has been an increasing dissatisfaction with RCTs in critical care because of their difficult implementation, the low number of positive trial results [18,19], and significant concerns regarding their generalizability to actual patients cared for in clinical practice [20]. Commentators have gone as far as to question the RCT’s role as the highest form of evidence in critical care [21], favoring a more measured balance between RCTs and well-conducted observational studies [22]. Our current study adds an important caveat to the ongoing conversation that weighs the relative value of observational studies and RCTs as clinical evidence [23]. We demonstrate that confounding by severity of illness is difficult to overcome without highly accurate risk adjustment, and that with large sample sizes there is greater risk of obtaining incorrect results. Prevailing wisdom suggests that when strong associations are measured in observational studies, they are unlikely to be fully attributed to confounding, as strong confounders are likely to be recognized, measured and controlled for in the analysis. However, our study suggests that in certain scenarios, even large effect sizes can be entirely due to confounding, even after risk-adjustment and despite only modest levels of confounding.

Highly accurate severity-of-illness scores have been developed for use in critical care, but these scores typically perform best when analyzing general ICU populations and are less reliable in patient subgroups. In a study of overall ICU mortality rates, acute physiology and chronic health evaluation (APACHE)-IV, simplified acute physiology (SAPS)-II, and mortality prediction model MPM had AUROC’s of 0.89, 0.87, and 0.81 respectively [24], values that would have reduced the risk of false positives in the current study. Even APACHE II performed well in a general ICU population (AUROC = 0.81) compared to the Charlson comorbidity index (AUROC = 0.63), a score that does not incorporate acute physiology parameters [25]. Yet, there are many examples of scores losing accuracy in particular patient populations, including patients with human immunodeficiciency virus [26], cardiogenic pulmonary edema [27], and trauma patients [28]; even APACHE-IV significantly under- or over-predicted mortality in 13% of medical conditions during its validation [10]. If authors report the AUROC of the risk adjuster in the patient sample they used during their particular analysis, it would help facilitate interpretation - our study examined internal AUROCs of the risk adjuster in the population studied, not the AUROCs in the original validation population.

The availability of larger and more detailed databases of critically ill adults is heralding a big-data revolution in critical care [6,29,30]. These highly granular clinical data might significantly bolster observational research in critical care by improving the accuracy of clinical measurement. Yet, the current study should remind researchers that studies utilizing large datasets are perhaps even more vulnerable to the fundamental problems of observational studies. As sample size increases, the ability to obtain results of high statistical significance increases, regardless of whether the results are real or biased. Thus, researchers must leverage these highly detailed clinical data to develop even more accurate methods of risk adjustment and to minimize unmeasured confounding for big data to genuinely revolutionize clinical research in critical care.

Our study should be interpreted in the context of several limitations. These results are simulated models of hypothetical observational studies, and not evidence that the result of any specific risk-adjusted observational study is confounded. Indeed, the extent of confounding cannot be measured or reported in any specific observational study. Whenever simulations are performed, choices must be made when modeling true events. In our simulations, we set the hypothetical treatment to be safe or beneficial, but more likely administered to patients with a higher baseline risk of death. Another scenario of interest is when a treatment with no effect on outcome is more likely administered to patients of lower risk. As this scenario is the symmetric inverse of what we studied, we suspect that similarly confounded studies with poor risk adjustment would falsely conclude the treatment was beneficial. The current study also only focused on problems with model discrimination (the risk adjuster accuracy), but poor model calibration could also cause problems in certain situations, especially if a study focused on a patient subgroup that was poorly predicted by the model. These simulations investigated confounding by severity of illness, where strong correlation between exposure, outcome and confounder led to large biases in effect estimates. Other forms of confounding, which may be less strongly correlated with exposure and outcome, would likely lead to less biased results.

Multiple factors besides risk adjustment accuracy should be considered when evaluating the validity of any observational research study. Study design is particularly important, as well-designed observational studies may be able to mitigate risk of confounding. By designing a study that makes comparisons between patients matched to be more similar, or situations where a physician does not choose a particular treatment based on an assessment of risk (particularly with natural experiments or instrumental variable designs that take advantage of the randomness induced by many styles of medical practice) confounding may be minimized.

## Conclusions

Our study demonstrates how confounding by severity of illness may be particularly problematic for observational studies in critical care. Even after employing conventional risk-adjustment, studies can obtain strikingly inaccurate results in certain circumstances. Providing the AUROC of the risk adjustment used on patients in the study may help assess a study’s risk of obtaining false positive results. Studies suspected to be at high risk for confounding based on clinical grounds should be interpreted cautiously, particularly for highly unexpected results.

## Key messages

- Confounding can lead to major errors in effect size estimates, making a safe treatment (OR for mortality = 1.0) appear harmful, and a beneficial treatment (OR for mortality <1.0) appear ineffective or harmful. Large treatment effects easily construed as true associations can be measured solely due to confounding, even in the presence of some degree of risk adjustment
- If study authors provide the AUROC of the risk adjustment used in the study analysis, it may help facilitate an evaluation of a study’s risk for confounding by severity of illness.
- Sample size increases the statistical significance of the results, whether confounded or not, thus larger sample sizes must be accompanied by better risk adjustment to prevent false discovery.
- The accuracy of risk adjustment at which an observational study was protected is cohort- and context-specific, and varied from >0.75 for some studies to >0.85 in studies with other distributions of risk.

## Additional file

Additional file 1:
**Supplemental analysis: when do confounding by indication and inadequate risk adjustment bias critical care studies?** This document provides the data used to create **Figure S2** and **Figure S3** in the main manuscript. It includes the results of simulations performed using the cohort of patients with bimodal distribution of risk. It also provides the sample code of the statistical simulations.

## Abbreviations

APACHE: acute physiology and chronic health evaluation
AUROC: area under the receiver operator characteristic curve
OR: odds ratio
RCT: randomized clinical trial
SAPS: simplified acute physiologic score
SOFA: sequential organ failure assessment
