# Supplementary material for: When do confounding by indication and inadequate risk adjustment bias critical care studies? A simulation study
Source: Crit Care. 2015 Apr 30;19(1):195. doi: 10.1186/s13054-015-0923-8 (PMC4432515; doi:10.1186/s13054-015-0923-8)
Supplement: Additional file 1: — Supplemental analysis: when do confounding by indication and inadequate risk adjustment bias critical care studies? This document provides the data used to create Figure S2 and Figure S3 in the main manuscript. It includes the results of simulations performed using the cohort of patients with bimodal distribution of risk. It also provides the sample code of the statistical simulations. [file 13054_2015_923_MOESM1_ESM.docx]

**Additional File 1**

**Supplemental Analysis: When do confounding-by-indication and inadequate risk adjustment bias critical care studies?**

Michael W. Sjoding, Kaiyi Luo, Melissa A. Miller, Theodore J. Iwashyna

Table S1 - Rates of falsely concluding a safe treatment (odds ratio = 1.0) caused statistically significant harm among non-surgical mechanical ventilation cohort studies

Table S2 - Rates of falsely concluding a beneficial treatment (odds ratio = 0.8) caused no benefit (false negative) or statistically significant harm (false harm) among non-surgical mechanical ventilation cohort studies

Table S3 - Rates of falsely concluding a safe treatment (odds ratio = 1.0) caused statistically significant harm among cohort studies from a population with a bimodal risk distribution

Table S4 – Rates of falsely concluding a beneficial treatment (odds ratio = 0.8) caused no benefit (false negative) or statistically significant harm (false harm) among cohort studies from a population with a bimodal risk distribution

Figure S1 – Distribution of baseline risk in the bimodal distribution of risk cohort

Figure S2 - Distribution of measured effect sizes among simulated studies when sample size was 1000 in the bimodal distribution of risk cohort in A) Low confounding scenarios and B) High confounding scenarios

Appendix – Simulation code (Stata 13)

Table S1 - Rates of falsely concluding a safe treatment (odds ratio = 1.0) caused statistically significant harm among non-surgical mechanical ventilation cohort studies

| Studies with n=1000 | |  |  |
| --- | --- | --- | --- |
| AUROC | No confounding | Low confounding | High confounding |
| 0.76 | 0.04 | 0.05 | 0.06 |
| 0.74 | 0.08 | 0.08 | 0.19 |
| 0.72 | 0.04 | 0.17 | 0.52 |
| 0.70 | 0.06 | 0.34 | 0.85 |
| 0.68 | 0.04 | 0.47 | 0.95 |
| 0.66 | 0.04 | 0.57 | 0.98 |
| 0.64 | 0.06 | 0.70 | 1.00 |
| 0.62 | 0.05 | 0.75 | 1.00 |
| 0.60 | 0.05 | 0.84 | 1.00 |
|  |  |  |  |
| Studies with n=10,000 | |  |  |
| AUROC | No confounding | Low confounding | High confounding |
| 0.76 | 0.06 | 0.04 | 0.06 |
| 0.74 | 0.06 | 0.35 | 0.87 |
| 0.72 | 0.06 | 0.88 | 1.00 |
| 0.70 | 0.06 | 1.00 | 1.00 |
| 0.68 | 0.05 | 1.00 | 1.00 |
| 0.66 | 0.05 | 1.00 | 1.00 |
| 0.64 | 0.06 | 1.00 | 1.00 |
| 0.62 | 0.05 | 1.00 | 1.00 |
| 0.60 | 0.04 | 1.00 | 1.00 |

Table S2 - Rates of falsely concluding a beneficial treatment (odds ratio = 0.8) caused no benefit (false negative) or statistically significant harm (false harm) among non-surgical mechanical ventilation cohort studies.

| Studies of n=1000 | | | | | |
| --- | --- | --- | --- | --- | --- |
|  | Low confounding | |  | High confounding | |
| AUROC | False Negative | False Harm |  | False Negative | False Harm |
| 0.76 | 0.67 | 0.00 |  | 0.72 | 0.00 |
| 0.74 | 0.84 | 0.00 |  | 0.94 | 0.01 |
| 0.72 | 0.92 | 0.01 |  | 0.90 | 0.09 |
| 0.70 | 0.94 | 0.04 |  | 0.60 | 0.40 |
| 0.68 | 0.93 | 0.07 |  | 0.36 | 0.64 |
| 0.66 | 0.90 | 0.10 |  | 0.17 | 0.83 |
| 0.64 | 0.82 | 0.18 |  | 0.07 | 0.93 |
| 0.62 | 0.75 | 0.25 |  | 0.04 | 0.96 |
| 0.60 | 0.68 | 0.32 |  | 0.01 | 0.99 |
|  |  |  |  |  |  |
| Studies of n=10,000 | | | | | |
|  | Low confounding | |  | High confounding | |
| AUROC | False Negative | False Harm |  | False Negative | False Harm |
| 0.76 | 0.00 | 0.00 |  | 0.01 | 0.00 |
| 0.74 | 0.14 | 0.00 |  | 0.76 | 0.00 |
| 0.72 | 0.70 | 0.00 |  | 0.51 | 0.49 |
| 0.70 | 0.93 | 0.06 |  | 0.00 | 1.00 |
| 0.68 | 0.71 | 0.29 |  | 0.00 | 1.00 |
| 0.66 | 0.32 | 0.68 |  | 0.00 | 1.00 |
| 0.64 | 0.10 | 0.90 |  | 0.00 | 1.00 |
| 0.62 | 0.01 | 0.99 |  | 0.00 | 1.00 |
| 0.60 | 0.00 | 1.00 |  | 0.00 | 1.00 |

Table S3 - Rates of falsely concluding a safe treatment (odds ratio = 1.0) caused statistically significant harm among cohort studies from a population with a bimodal risk distribution

| Studies with n=1000 | | | |
| --- | --- | --- | --- |
| AUROC | No confounding | Low confounding | High confounding |
| 0.84 | 0.06 | 0.096 | 0.287 |
| 0.8 | 0.052 | 0.672 | 0.998 |
| 0.76 | 0.048 | 0.949 | 1 |
| 0.72 | 0.042 | 0.997 | 1 |
| 0.6 | 0.047 | 1 | 1 |
|  |  |  |  |
| Studies with n=10,000 | | | |
| AUROC | No confounding | Low confounding | High confounding |
| 0.84 | 0.052 | 0.532 | 0.987 |
| 0.8 | 0.045 | 1 | 1 |
| 0.76 | 0.044 | 1 | 1 |
| 0.72 | 0.053 | 1 | 1 |
| 0.6 | 0.048 | 1 | 1 |

Table S4 – Rates of falsely concluding a beneficial treatment (odds ratio = 0.8) caused no benefit (false negative) or statistically significant harm (false harm) among cohort studies from a population with a bimodal risk distribution

| Studies of n=1000 | | | | | |
| --- | --- | --- | --- | --- | --- |
|  | Low confounding | |  | High confounding | |
| AUROC | False Negative | False Harm |  | False Negative | False Harm |
| 0.84 | 0.93 | 0.00 |  | 0.95 | 0.04 |
| 0.8 | 0.76 | 0.24 |  | 0.03 | 0.97 |
| 0.76 | 0.25 | 0.75 |  | 0.00 | 1.00 |
| 0.72 | 0.06 | 0.94 |  | 0.00 | 1.00 |
| 0.6 | 0.00 | 1.00 |  | 0.00 | 1.00 |
|  |  |  |  |  |  |
| Studies of n=10,000 | |  |  |  |  |
|  | Low confounding | |  | High confounding | |
| AUROC | False Negative | False Harm |  | False Negative | False Harm |
| 0.84 | 0.60 | 0.00 |  | 0.86 | 0.14 |
| 0.8 | 0.02 | 0.99 |  | 0.00 | 1.00 |
| 0.76 | 0.00 | 1.00 |  | 0.00 | 1.00 |
| 0.72 | 0.00 | 1.00 |  | 0.00 | 1.00 |
| 0.6 | 0.00 | 1.00 |  | 0.00 | 1.00 |

Figure S1 – Distribution of baseline risk in the bimodal distribution of risk cohort

Figure S2 - Distribution of measured effect sizes among simulated studies when sample size was 1000 in the bimodal distribution of risk cohort in A) Low confounding scenarios and B) High confounding scenarios

A) Low Confounding

B) High Confounding

e-Appendix – Simulation code (Stata 13)

*****

* this file runs simulations with the non-surgical mechanical ventilation cohort

* saving each simulation run as run_`number' and a summary

* file of the simulations summary is sim_totaler_NPOV

cd "/Users/Mike/Documents/Confounding by Indication Paper/Results"

set more off

capture program drop confounder

program confounder, rclass

version 10.1

syntax [,cases(integer 100000) rasd(real 0.3) sw(real 1) dor(real 1)]

if `cases' < 10 {

display as error ///

"Must have more than 10 cases to run this"

error 198

}

if `rasd' <= 0 {

display as error ///

"Risk-adjuster Standard Deviation must be strictly positive"

error 198

}

if `sw' < 0 {

display as error ///

"Sickness Weight must be non-negative"

error 198

}

if `dor' <= 0 {

display as error ///

"Drug Effect Odds Ratio must be strictly positive"

error 198

}

drop _all

*** these are the basic parameters for the simulations

scalar risk_adjuster_sd = `rasd'

scalar sickness_weight = `sw'

scalar drug_dose_effect_odds_ratio = `dor'

*** open the patient data file used during simulations

use riskfile_npov, clear

***draw a sample with replacement of defined size

bsample `cases'

*** generate the "risk-adjuster" - an unbiased estimate of the

*** baseline probability of death (the variable truesickness)

gen risk_adjuster_error = rnormal(0,risk_adjuster_sd)

gen risk_adjuster = truesickness + risk_adjuster_error

*** determine the probability of getting the drug, which is a weighted s

*** sum of a random component and baseline probability of death,

*** normalized to stay 0 to 1

*** Each person has a random threshold above which they get the drug

*** and the sickness_weight increases the degree to which their

*** drug receipt is correlated with truesickness

gen drug_threshold = runiform()

gen random_component_getting_drug = runiform()

gen total_prob_getting_drug = (random_component_getting_drug + ///

sickness_weight*truesickness) / (1 + sickness_weight)

gen got_drug = (total_prob_getting_drug > drug_threshold)

*** Tally of how many received the drug

count if got_drug==1

return scalar n_got_drug=r(N)

*** Tally of how many didn't receive the drug

count if got_drug==0

return scalar n_no_drug=r(N)

gen drug_effect_odds_ratio = got_drug * drug_dose_effect_odds_ratio

*** Bernoulli trial to determine if patient dies

gen outcome_threshold = runiform()

gen dead_nodrug = (truesickness > outcome_threshold)

**** This section of the code

****examines the impact on the measured odds ratio

**** if the drug has a true effect on outcomes

gen odds_truesickness = truesickness / (1-truesickness)

gen odds_sickness_drug = odds_truesickness * drug_effect_odds_ratio ///

if got_drug==1

replace odds_sickness_drug = odds_truesickness if got_drug==0

gen probability_sickness_drug = odds_sickness_drug / ///

( 1 + odds_sickness_drug)

gen dead_w_drug = (probability_sickness_drug > outcome_threshold)

*** Determine how good is our risk-adjuster?

quietly logit dead_nodrug risk_adjuster

lroc, nograph

scalar dead_risk_adjuster_auroc = r(area)

*** How much confounding of drug receipt by true sickness is there?

*** for ease of exposition, we rescale truesickness

gen true_sick_dec = truesickness * 10

quietly logit got_drug true_sick_dec

scalar drug_sick_beta = _b[true_sick_dec]

scalar drug_sick_or = exp(drug_sick_beta)

*** What is the apparent relationship between the drug and

*** the risk-adjuster?

quietly logit got_drug risk_adjuster

lroc, nograph

scalar drug_risk_adjuster_auroc = r(area)

*** what is the apparent measured drug effect?

quietly logit dead_w_drug risk_adjuster got_drug

scalar got_drug_beta = _b[got_drug]

scalar got_drug_or = exp(got_drug_beta)

scalar got_drug_se = _se[got_drug]

scalar got_drug_z = got_drug_beta / got_drug_se

scalar got_drug_p = 2 * normprob(-abs(got_drug_z))

*** return the scalars

return scalar total_cases = _N

return scalar ra_sd = risk_adjuster_sd

return scalar sick_wgt = sickness_weight

return scalar drug_or_real_return = drug_dose_effect_odds_ratio

return scalar dead_ra_auroc_return = dead_risk_adjuster_auroc

return scalar drug_ra_auroc_return = drug_risk_adjuster_auroc

return scalar drug_or_measured_return = got_drug_or

return scalar drug_beta_return = got_drug_beta

return scalar drug_se_return = got_drug_se

return scalar drug_p_return = got_drug_p

return scalar significant_return = (got_drug_p < 0.05)

return scalar drug_sick_or_return = drug_sick_or

end

set seed 11101969

postfile sim n n_drug n_nodrug sw risk_sd drug_or_real dead_ra_auroc ///

drug_ra_auroc drug_or_measured drug_p_measured drug_significant ///

drug_sick_or using sim_totaler_npov, replace

*** We will simulate n=1000, n=10,000 cases

scalar counter=0

foreach x_cases in 1000 10000 {

** We will consider cases in which there is no, little, or much confounding]

foreach x_sw in 0 1.1 10 {

** We will create risk-adjusters that vary in their mean accuracy from good to poor

foreach x_rasd in 0.02 0.12 0.2 0.31 0.47 {

di "Cases: "`x_cases' " RA_sd:" `x_rasd' " SW:" `x_sw'

scalar counter=counter+1

local i2 = counter

simulate cases=r(total_cases) ///

cases_drug=r(n_got_drug) ///

cases_nodrug=r(n_no_drug) ///

risk_sd=r(ra_sd) ///

sick_weight=r(sick_wgt) ///

drug_or_real=r(drug_or_real_return) ///

dead_ra_auroc=r(dead_ra_auroc_return) ///

drug_ra_auroc=r(drug_ra_auroc_return) ///

drug_or_measured=r(drug_or_measured_return) ///

drug_p_measured=r(drug_p_return) ///

drug_significant=r(significant_return) ///

drug_sick_or=r(drug_sick_or_return) ///

, saving(run_`i2', replace) ///

reps(1000): confounder, cases(`x_cases') rasd(`x_rasd') ///

sw(`x_sw') dor(1)

mean cases cases_drug cases_nodrug sick_weight risk_sd drug_or_real ///

dead_ra_auroc drug_ra_auroc drug_or_measured ///

drug_p_measure drug_significant drug_sick_or

post sim (_b[cases]) (_b[cases_drug]) (_b[cases_nodrug]) ///

(_b[sick_weight]) (_b[risk_sd]) (_b[drug_or_real]) ///

(_b[dead_ra_auroc]) (_b[drug_ra_auroc]) (_b[drug_or_measured]) ///

(_b[drug_p_measure]) (_b[drug_significant]) (_b[drug_sick_or])

}

}

}

postclose sim

use sim_totaler_npov, clear

list
